# Lifetime Prevalence and Correlates of Patient-Perceived Medical Errors Experienced in the U.S. Ambulatory Setting: A Population-Based Study

**DOI:** 10.1089/heq.2020.0009

**Published:** 2020-10-07

**Authors:** David N. Sundwall, Mark A. Munger, Casey R. Tak, Mike Walsh, Michael Feehan

**Affiliations:** ^1^ Department of Family and Preventative Medicine, University of Utah, Salt Lake City, Utah, USA.; ^2^ Department of Pharmacotherapy, University of Utah, Salt Lake City, Utah, USA.; ^3^ Department of Internal Medicine, University of Utah, Salt Lake City, Utah, USA.; ^4^ Division of Pharmaceutical Outcomes and Policy, Eshelman School of Pharmacy, The University of North Carolina at Chapel Hill, Chapel Hill, North Carolina, USA.; ^5^ Hall Partners, Ltd., New York, New York, USA.; ^6^ Kantar, San Francisco, California, USA.

**Keywords:** ambulatory care, medical errors, U.S. health care

## Abstract

**Background:** The rate of safety harm self-perceived medical errors and harms reported in the U.S. ambulatory system is not well characterized.

**Objectives:** To determine the prevalence of U.S. adult ambulatory care patient self-perceived safety harms and to gauge the degree of association between harms with various patient characteristics and outcomes.

**Methods:** A large U.S. cross-sectional online survey of 9206 ambulatory care adults was assessed for their perception of medical errors and harms during care (misdiagnosis, mistakes in care, and wrong or delayed treatment) and also included patient demographics, health status, comorbidities, insurance status, income, barriers to care (affordability, transportation, and family and social support), number of visits to primary health care services in the past 12 months, and use of urgent or emergency care in the last 12 months.

**Results:** The overall rate of self-perceived medical errors and harms among adult patients in the ambulatory care setting was 36%. Female patients, independent of age, and those with multiple comorbidities or barriers to care, reported the highest number of medical errors. Utilization of multiple providers was associated with a greater number of reported medical errors, often resulting in changing health care providers. Patients who reported having trouble affording health care or navigating the system to receive care also reported higher levels of harm. They were cared for by multiple providers, often switch providers, and their care is associated with greater utilization of health care resources. Patients reporting the highest rates of harm had greater use of hospital and emergency room care.

**Conclusions:** This large U.S. adult ambulatory care study provides evidence that patient self-perceived medical errors and harms reported by patients are common. Patient self-perceived medical errors and harms occur most commonly in women, with poor health, limitation of activities, and who have three or more comorbidities.

## Introduction

The bulk of health care is delivered through the ambulatory care setting. Approximately 1 billion patient care visits occur each year in the United States, far outpacing hospital discharges.^1,2^ Insufficient monitoring of patient safety may be more evident in the ambulatory care system compared with the institutional setting, thereby placing patients at increased risk of harm.^3^ Reasons for an ambulatory care safety disparity may include a wide variety of decentralized settings, lack of connectivity of electronic health records between providers, as well as a greater need for patients to engage in more active participation to receive care.^4^ As patients seek to take greater control of their own health care decisions, more attention to the patient experience needs to occur, safety being one dimension of a quality care experience.

Ambulatory care patient safety practice research has principally been focused on the patient/health care team interface and has not been specific to the patient-reported experience. Literature has focused on systems issues such as misdiagnosis,^5,6^ transitions of care,^7^ test result management,^8^ and medication complexity including prescribing, dispensing, monitoring, and identifying drug interactions,^9–11^ medication nonadherence,^12,13^ and communication lines between provider and patient.^6,14^ Identifying patient characteristics associated with patient-perceived medical errors while navigating through the ambulatory health care system is important to understand.^4,15,16^

This prospective large national study was undertaken to understand adult patient self-perceived experience with medical errors and harm resulting from ambulatory care settings through an online survey, using a published set of quantitative questions categorizing medical errors.^5^ The lifetime and 12-month prevalence of patient self-perceived medical errors was then associated with patient characteristics, including demographics, health status, comorbidities, insurance status, income, barriers to care (affordability, transportation, and family and social support), number of visits to primary health care services in the past 12 months, and use of urgent or emergency care in the last 12 months.

## Methods

This study was approved by the University of Utah Institutional Review Board and was conducted in accordance with the World Medical Association Declaration of Helsinki: Ethical Principles for Medical Research Involving Human Subjects.

### Sample

The study involved a self-administered online survey that was made available to a random sample of patients across the United States. The patient sample comprised 10,006 adults recruited from an established nationally representative panel of individuals in the United States, who opted-in to be contacted for research purposes (Universal Survey Center, Inc., d/b/a SHC Universal New York, NY). Panelists accessed the survey electronically, through a link in an invitation e-mail, which offered a minimal honorarium for participation. Respondents were prescreened to be adults aged 18 years or older. A total of 75,908 e-mail invitations were sent out randomly between August 27, 2015, and September 21, 2015; 48,959 participants responded (64.5%), 15,572 (31.8%) of whom were qualified for participation by meeting the inclusion criteria below. The data from the 10,006 who completed the survey were analyzed for quality, and data were removed from the data set for inconsistent responses, resulting in a final total of 9202 patients with data for analyses.

Patients accessed the survey electronically, through a link in the invitation e-mail. In the invitation, patients were offered a minimal honorarium for their participation. The study sponsor was not revealed to the participants at any point during the survey to prevent response bias.

Patients were prescreened to participate if they met the following age-specific inclusion criteria: aged 18 years or older. Patients with Veterans Affairs, CHAMPUS, or TRICARE insurance or who received care through Kaiser, Kaiser Permanente, the Permanente, or the Permanente Medical Group were excluded. Quotas were set to approximate the 2014 U.S. Census Bureau^17^ adult population that included women, various ethnic minorities, lower income populations, and across geographies (e.g., rural vs. urban/suburban). The proportion with low income was defined as the difference between the poverty threshold and 200% above the poverty threshold.^18^

### Questionnaire

Survey questions regarding medical errors were taken from published reports of patient perceptions of medical errors in the ambulatory care setting.^5^

Each participant was asked questions regarding his or her perceptions of medical errors he or she could recall over the lifetime (ever made) or in the last 12 months. Participants were not given a definition of a medical error.^5^

(1) Has a doctor in a doctor's office ever made a mistake in your care? If YES, has this happened in the last 12 months?
(2) Has a doctor in a doctor's office ever made a wrong diagnosis or misdiagnosed you? If YES, has this happened in the last 12 months?
(3) Has a doctor in a doctor's office ever given you the wrong medical treatment or delayed treatment? If YES, has this happened in the last 12 months?

For each of the three questions above, a follow-up question was asked: “How much harm did this cause you?”

A lifetime measure of any patient-perceived medical error and harm, defined as a positive response to any of the medical error questions (i.e., mistake in care, wrong diagnosis or misdiagnosis, or wrong medical treatment or delayed treatment), was calculated for the entire sample. A separate measure for patient self-perceived medical errors or harms in the last 12 months, defined as a positive response to any of the medical error questions and an indication that this happened within the last 12 months, was calculated for the entire sample.

Additional questions were designed to focus on the patients' demographic characteristics, health status, interactions with the health care system, and therapeutic management. These included gender, race, ethnicity, age, geographies (e.g., rural vs. urban, region in the United States), income (poverty, low, medium, or high income level), insurance status, number of current comorbidities aggregated into a continuous value, current perceived health status, ability to afford care and medication costs, transportation issues, availability of support system(s), number of visits to primary health care services in the past 12 months, primary location utilized for health care needs, and use of urgent or emergency care in the last 12 months. Those living in poverty were determined by age and number of children in the home.

### Analytical methods

All analytical methods were prespecified before conducting the survey. Frequencies and means for categorical and continuous variables, respectively, described survey responses. Bivariate logistic regression models were conducted to identify factors from the survey that were significantly associated with the composite measure of medical errors ever experienced (lifetime) or, separately, experienced in the previous 12 months. Specifically, we examined the impact that demographic characteristics, health status, interactions with the health care system, and therapeutic management had on self-reported medical errors. Multivariable logistic regression models, including all survey variables from the bivariate analyses, were used to identify the characteristics most associated with lifetime or previous 12 months self-reported medical errors. The level of significance was determined *a priori* to be α < 0.05. All analyses were completed using SAS v9.4 (SAS Institute, Cary, NC).

## Results

### Patient characteristics and health care services utilization

Adult participants (9206) were middle aged (50.2±15.1 years), and the majority were female (54%) (Table 1). Approximately 20% were aged 65 years or older. Those identifying as Hispanic comprised 12% of the sample, and 11% identified as African American. The majority (94.6%) were insured.

**Table 1.** Demographic Characteristics of 9202 Adults from the General Population Surveyed About Primary Health Care Medical Errors

Characteristic	Total	Male	Female
	n=9202	n=4226 (45.9%)	n=4976 (54.1%)
	n (%)	n (%)	n (%)
Age			
Age 65+	1688 (18.3)	830 (19.6)	858 (17.2)
18–64	7514 (81.7)	3396 (80.4)	4118 (82.8)
Hispanic origin			
Hispanic origin	1088 (11.8)	569 (13.5)	519 (10.4)
Non-Hispanic origin	8114 (88.2)	3657 (86.5)	4457 (89.6)
Race			
African American	985 (10.7)	453 (10.7)	532 (10.7)
Non-African American	8217 (89.3)	3773 (89.3)	4444 (89.3)
Insurance status			
Insured	8701 (94.6)	4008 (94.8)	4693 (94.3)
Noninsured	501 (5.4)	218 (5.2)	283 (5.7)
Poverty level^a^			
Poverty	1346 (14.6)	447 (10.6)	899 (18.1)
Low income	2229 (24.2)	916 (21.7)	1313 (26.4)
Above low income	5627 (61.1)	2863 (67.7)	2764 (55.5)
Income			
Very low (less than $25,000)	2171 (23.6)	817 (19.3)	1354 (27.2)
Low ($25,000–$49,999)	2790 (30.3)	1182 (28.0)	1608 (32.3)
Medium ($50,000–$99,999)	2990 (32.5)	1522 (36.0)	1468 (29.5)
High ($100,000 and above)	1251 (13.6)	705 (16.7)	546 (11.0)
Community residence			
Rural	1748 (19.0)	690 (16.3)	1058 (21.3)
Small city or town	2879 (31.3)	1311 (31.0)	1568 (31.5)
Suburb of a large city	3203 (34.8)	1525 (36.1)	1678 (33.7)
Large city	1372 (14.9)	700 (16.6)	672 (13.5)
Region			
Northeast	1759 (19.1)	838 (19.8)	921 (18.5)
Midwest	2273 (24.7)	1025 (24.3)	1248 (25.1)
South	3546 (38.5)	1560 (36.9)	1986 (39.9)
West	1624 (17.6)	803 (19.0)	821 (16.5)

^a^ Poverty threshold was determined by age, number of children in the home, and income as defined by the U.S. Census Bureau for 2014 (https://www.census.gov/hhes/www/poverty/data/threshld/). Low income was defined as between the poverty threshold and 200% above the poverty threshold (https://www.census.gov/hhes/www/poverty/methods/definitions.html). All others were considered above low income.

Those living in poverty represented 14.6% of the sample. The proportion with low income was 24.2%. About 65% lived in a large city suburb or small city or town, with 19% living in rural settings. The southern region had the largest number of participants.

Patients' perception of their general health was between fair to very good. The proportion of patients who rated their health as excellent was 4%, and rating their health as poor was 5%. The overall comorbidity average score was 2.7±2.1. Approximately 50% visited a primary care provider every 6 months to 1 year, with about 40% visiting two to five times in the past year. Only 5% of patients did not have a primary care provider. The most frequent provider was a primary care physician, and patients visited them on an average of 3.4 times in the past year, followed by a community pharmacist at 2.5 times in the past year, and a specialist visited twice in the past year. Urgent care was utilized by less than 1% in the past year.

### Prevalence of medical errors and covariates

The composite lifetime prevalence of patient-perceived medical errors based on who answered yes to any of the three key questions was 36%, with 10.5% occurring in the last 12 months (Table 2). Patient-perceived medical errors were stated by 25% who recalled that a doctor had ever made a mistake in their care. A misdiagnosis was the most common medical error at 26%. Of those, 23% reported misdiagnosis occurring in the past 12 months. Of the patients who had stated a misdiagnosis occurred, 21% reported harm, 54% a little or some harm, 16% a lot, and 8% severe. A wrong medical treatment or delayed treatment ever experienced was perceived by 17%; 31% had the experience in the past 12 months. Sixty-five percent indicated they were not adversely affected by a wrong or delayed treatment; however, 23% stated they were harmed a lot, and 12% reported suffering a severe outcome. Twenty-three percent changed doctors related to a perception of a wrong diagnosis or wrong treatment, primarily occurring in the last 12 months.

**Table 2.** Frequency of Self-Reported Medical Errors by Type

Variable n=9202	Frequency (%) EVER	Frequency (%) last 12 months
(A) Has a doctor in a doctor's office EVER made a mistake in your care?	2298 (25.0)	
(B) Has a doctor in a doctor's office EVER made a wrong diagnosis or misdiagnosed you?	2401 (26.1)	
(C) Has a doctor in a doctor's office EVER given you the wrong medical treatment or delayed treatment?	1813 (19.7)	
Composite of A, B, or C	3305 (35.9)	
Composite of A, B, or C within previous 12 months	969 (10.5)	

The source of care delivery, whether a primary care or specialist care physician practice, nurse practitioner, physician assistant, or community pharmacy, was not related to a patient-perceived medical error.

Patient characteristics found to be associated with increasing odds of a lifetime patient self-perceived medical errors are reported in Table 3. Females reported the highest rate of medical errors with an adjusted odds ratio (AOR) of 1.44 (95% confidence interval [CI]: 1.26–1.65). Hispanic ethnicity and African American race were associated with a lower AOR for perceived medical errors. Age ≥65 years was also associated with a lower AOR. Adjusted results indicate no general trend in the relationship between income or difficulty paying for health care services and patient-perceived medical errors. Persons living in the Midwest or West had higher AOR of patient-perceived medical errors compared with persons living in the East.

**Table 3.** Bivariate and Multivariable Regression Analysis: Patient, Health, and Utilization Characteristics Associated with Patient-Perceived Lifetime Medical Errors in the U.S. Ambulatory Care System

Patient characteristics	Bivariate association	Multivariable association
	OR and 95% CI	AOR and 95% CI^a^
Female	1.54 (1.41–1.68)	1.42 (1.29–1.56)
Hispanic	0.84 (0.74–0.97)	0.84 (0.71–0.98)
African American	0.66 (0.56–0.76)	0.62 (0.53–0.73)
Age ≥65 years	0.73 (0.65–0.82)	0.73 (0.64–0.83)
Poverty^b^	1.48 (1.31–1.67)	—
Low income^c^	1.30 (1.18–1.44)	—
Income level (reference=high income)		
Very low income (<$25,000/year)	1.42 (1.23–1.64)	—
Low income (≥$25,000 to $49,999)	1.12 (0.98–1.30)	0.84 (0.70–0.99)
Medium income ($50,000 to $99,999)	0.97 (0.84–1.12)	—
No insurance	1.31 (1.09–1.57)	—
Type of community (Reference=suburb)		
Rural	1.13 (0.89–1.14)	**—-**
Small city or town	1.14 (1.03–1.23)	**—-**
Large city	0.94 (0.82–1.07)	**—-**
Region (Reference=east)		
South	1.17 (1.02–1.33)	—
Midwest	1.26 (1.12–1.42)	1.24 (1.09–1.42)
West	1.36 (1.18–1.56)	1.41 (1.20–1.64)
Health (Reference=excellent)		
Poor	3.15 (2.31–4.28)	1.42 (1.01–1.98)
Fair	2.63 (2.02–3.43)	1.46 (1.09–1.94)
Good	1.65 (1.28–2.14)	**—-**
Very good	1.34 (1.03–1.75)	**—-**
Activities limited by health (Reference=no)		
A little bit	1.85 (1.67–2.04)	1.38 (1.24–1.55)
Lot	2.24 (2.00–2.50)	1.32 (1.15–1.52)
Comorbidities (reference=0)		
1 or 2	1.06 (0.91–1.23)	**—-**
>2	1.97 (1.71–2.28)	1.35 (1.15–1.58)
Comorbidity score	1.19 (1.17–1.22)	**—-**
Difficulty with health care cost	1.21 (1.05–1.37)	**—-**
Difficulty with transportation to medical care	1.26 (1.08–1.41)	**—-**
Utilized >2 providers/last 12 months	2.08 (1.90–2.26)	1.54 (1.39–1.71)
Utilized ≥2 locations for primary care services/last 12 months	2.03 (1.84–2.23)	1.37 (1.22–1.55)
Number of visits to primary provider (Reference=every month)		
Every 2–5 months	0.78 (0.67–0.90)	**—-**
Every 6 months	0.57 (0.49–0.66)	**—-**
Yearly	0.56 (0.48–0.66)	**—-**
Every few years	0.73 (0.54–0.99)	**—-**
Received most care at hospital/urgent care	1.55 (1.31–1.83)	1.26 (1.04–1.54)
Emergency care visits 1–2 times/last 12 months	1.72 (1.57–1.90)	**—-**
Emergency care visits ≥3 times/last 12 months	2.83 (2.37–3.39)	1.34 (1.08–1.66)

^a^ Adjusted for additional variables not shown: Difficulty paying for medication cost and has support of others.

^b^ Poverty threshold was determined by age, number of children in the home, and income as defined by the U.S. Census Bureau for 2014 (https://www.census.gov/hhes/www/poverty/data/threshld/).

^c^ Low income was defined as between the poverty threshold and 200% above the poverty threshold (https://www.census.gov/hhes/www/poverty/methods/definitions.html). All others were considered above low income.

AOR, adjusted odds ratio; CI, confidence interval; OR, odds ratio.

Decreased health and increased utilization of health care services were also associated with lifetime patient-perceived medical errors (Table 3). Patients who rated their health as poor or only fair were more likely to report a medical error (AOR 1.42 [95% CI: 1.01–1.98] and 1.46 [95% CI: 1.09–1.94]). In addition, limited activity due to health was associated with a greater rate of perceived medical errors. Patients with more than 2 comorbidities had an AOR of 1.35 (95% CI: 1.15–1.58). Patients utilizing more than two providers or were receiving care at more than two provider locations in the last 12 months had higher AORs (1.54 [95% CI: 1.39–1.71] and 1.37 [95% CI: 1.22–1.55], respectively). An emergency care visit frequency of three or more times in the past 12 months was associated with an elevated medical error rate, with an AOR of 1.34 (95% CI: 1.08–1.66).

Similar associations were seen with patient-perceived medical errors in the previous 12 months (Table 4). The largest predictors of patient-perceived medical errors were visiting emergency care three or more times in the previous 12 months (AOR 3.73 [95% CI: 2.91–4.78]), visiting emergency care one to two times in the previous 12 months (AOR 1.82 [95% CI: 1.53–2.16]), utilizing more than two providers in the last 12 months (AOR 1.53 [95% CI: 1.29–1.82]), and visiting a primary provider less frequently than each month.

**Table 4.** Bivariate and Multivariable Regression Analysis: Patient, Health, and Utilization Characteristics Associated with Patient-Perceived Medical Errors in the Previous 12 Months in the U.S. Ambulatory Care System

Patient characteristics	Bivariate association	Multivariable association
	OR and 95% CI	AOR and 95% CI^a^
Female	1.44 (1.26–1.65)	1.37 (1.18–1.59)
Hispanic	1.13 (0.93–1.38)	**—-**
African American	0.92 (0.73–1.15)	**—-**
Age ≥65 years	0.64 (0.53–0.78)	0.79 (0.64–0.98)
Poverty^b^	1.76 (1.47–2.10)	**—-**
Low income^c^	1.45 (1.24–1.70)	**—-**
Very low income (< $25,000/year)	0.79 (0.67–0.94)	**—-**
Low income (≥ $25,000 to $49,999)	0.60 (0.50–0.72)	**—-**
Medium income ($50,000 to $99,999)	0.62 (0.49–0.78)	**—-**
No insurance	0.86 (0.65–1.13)	**—-**
Type of community (Reference=suburb)		
Rural	0.98 (0.81–1.18)	**—-**
Small city or town	0.91 (0.75–1.09)	**—-**
Large city	0.99 (0.79–1.25)	**—-**
Region (Reference=east)		
South	0.91 (0.74–1.13)	—-
Midwest	1.14 (0.95–1.38)	—-
West	1.31 (1.06–1.63)	1.33 (1.06–1.68)
Health (Reference=excellent)		
Poor	2.11 (1.36–3.26)	**—-**
Fair	1.70 (1.15–2.50)	—-
Good	1.01 (0.68–1.48)	**—-**
Very good	0.80 (0.54–1.20)	**—-**
Activities limited by health (Reference=no)		
A little bit	2.09 (1.79–2.45)	1.32 (1.11–1.59)
Lot	2.60 (2.20–3.08)	**—-**
Comorbidities (Reference=0)		
1 or 2	1.46 (1.10–1.94)	**—-**
>2	2.81 (2.14–3.69)	**—-**
Comorbidity score	1.21 (1.18–1.25)	**—-**
Difficulty with health care cost	1.84 (1.61–2.10)	1.37 (1.14–1.63)
Difficulty with transportation to medical care	2.01 (1.75–2.29)	1.31 (1.10–1.57)
Utilized >2 providers/last 12 months	3.00 (2.60–3.47)	1.53 (1.29–1.82)
Utilized ≥2 locations for primary care services/last 12 months	3.00 (2.62–3.44)	1.37 (1.16–1.62)
Number of visits to primary provider (Reference=every month)		
Every 2–5 months	0.54 (0.45–0.65)	**—-**
Every 6 months	0.31 (0.25–0.39)	0.69 (0.54–0.87)
Yearly	0.25 (0.20–0.33)	0.61 (0.46–0.82)
Every few years	0.20 (0.11–0.38)	0.42 (0.22–0.81)
Location that received most care hospital/urgent care	2.14 (1.72–2.67)	1.33 (1.04–1.70)
Emergency care visits 1–2 times/last 12 months	3.05 (2.63–3.54)	1.82 (1.53–2.16)
Emergency care visits ≥3 times/last 12 months	8.39 (6.83–10.31)	3.73 (2.91–4.78)

^a^ Adjusted for additional variables not shown: Difficulty paying for medication cost and has support of others.

^b^ Poverty threshold was determined by age, number of children in the home, and income as defined by the U.S. Census Bureau for 2014 (https://www.census.gov/hhes/www/poverty/data/threshld/).

^c^ Low income was defined as between the poverty threshold and 200% above the poverty threshold (https://www.census.gov/hhes/www/poverty/methods/definitions.html). All others were considered above low income.

## Discussion

The majority of health care is delivered through the ambulatory care setting where there remains a small literature base for patient self-perception medical errors and harms. This large U.S. national survey of adults who self-perceived medical errors in ambulatory health care settings shows that they are common. Women, independent of age, reported a greater likelihood of patient-perceived medical errors. Patients who have difficulty paying for medical care, who have lower levels of perceived health status, who have limitation of activity, or who report >2 comorbidities have higher odds of perceptions of a medical error. Utilization of multiple providers is associated with greater risk of the perception of a medical error. These patients utilize higher levels of health care resources, such as a frequent of emergency care visits. The ability to pay for health care is associated with the rates of a self-perceived medical error in the ambulatory care setting.

The results of this study show the rate of a patient-perceived medical error to be comparable with those reported elsewhere. A U.S. survey research has reported wide rates of ambulatory care medical errors ranging from 11% to as high as 53%.^5–6,10,19–23^ Across the world, rates of survey-reported medical errors are 5% in Latin America, 1–2% in the United Kingdom, and as high as 37.3% in Alberta, Canada.^15,24,25^ In Malaysia, among 12 privately funded primary care clinics involving 1753 medical records randomly selected, diagnostic errors were common at 53.2% attributed to management errors methodologically defined as an error in investigation, medication, or in the decision-making process.^23^ The authors stated that ∼40% of the errors had the potential for harm.^23^ Many sampling approaches have been used to ascertain the level of medical errors, all of which suffer from potential bias; however, studies over time have found that patient reporting is reliable, but may need further evaluation to confirm validity.^21,26,27^

Perception of an error in medical management compared with progression of the natural history of disease still remains unknown. One common theme noted is that the majority of medical errors reported are not perceived as severe.^15,28^ The elderly appear to be most likely affected by medical errors^15^; however, this has not been a consistent finding.^5^ Our data do not confirm that the elderly are more likely to suffer a medical error from their interaction with the U.S. health care system. The reason for this outcome is not readily apparent from our data, but patient demographic mix associated with the frequency of visits, most having a primary care provider, may have contributed to this finding.

The ambulatory care setting offers complexity that may contribute to a higher risk of medical errors. A recent narrative review suggests that the World Health Organization leadership considers bringing together a multidisciplinary effort to address the common challenges and opportunities to reduce diagnostic errors.^29^ Complexity may be due to a lack of a coordinated data management and reporting through many different electronic medical record platforms, although adoption of health information technology is improving rapidly.^15,30^ Time between provider visits and short visit time in the provider/patient relationship may reduce patient connectivity and satisfaction, thereby contributing to safety disparities.^30^ These time issues can lead to misdiagnosis, unnecessary referrals, overtreatment, and patient self-management of their condition, including whether to initiate, adjust, continue, or discontinue care, often without provider input. Health illiteracy, affecting as many as 50% of the population, must also should be considered.^31^ Ambulatory care complexity reflects how important the provider/patient interaction is to patient safety in the ambulatory setting.^14^ Collecting information from patients about their perceptions of the current provider/patient relationship could assist in greater understanding of why patients switch providers as a result of their perceived medical errors.^32^

Our results show that persons often considered disadvantaged in health care, namely minorities and the poor, were not disproportionately suffering medical errors in the ambulatory setting. This finding is in agreement with studies suggesting patient perceptions of medical errors are true events.^5^ Reporting of medical errors is not reflective of ethnicity, but rather a patient's satisfaction with care.^16,33,34^ However, if those persons were suffering from poor health, have limitation of activity, have multiple comorbidities, have more than two providers, or were disadvantaged from being able to afford or access health care, they had a higher likelihood of experiencing a medical error.

Our results support the need for a higher level of conscious of physician oversight throughout the primary care setting, potentially through system alerts to mitigate the potential for medical errors. To this end, patients may be monitored through a coordinated ambulatory care team for assistance with diet, transportation issues that focus on the ability to meet appointments, especially transitions between providers and obtaining medications on a regular basis, physical therapy, hearing problems, enriching their social network, and monitoring depression and anxiety.

Ambulatory care medical errors are likely to be associated with higher health care costs. Patients with poor health, lack of access, or multiple comorbidities show an increased use of urgent care, emergency care visits, and hospitalizations in our study. One view of this study result is patients who have trouble negotiating the ambulatory health care system may experience medical errors and go on to develop more serious health care problems, requiring higher levels of care. Addressing ambulatory care safety would be cost effective.

This study is subject to some limitations. The data available for this analysis were cross sectional; therefore, no causal relationship between the correlates and medical errors can be established. The study is limited by the following groups that could not be surveyed: patients who have died, were hospitalized, became senile, developed significant mental or behavioral health issues, or acquired a disability that limited Internet use would all likely be excluded. Patients who have limited ability to use computers also limited the study population. We surveyed only adult patients who had utilized the ambulatory health care system; thus, the results do not reflect pediatric patient medical errors. The results principally apply to patients who were insured and may not reflect patient perceptions of medical errors in those without health care insurance. The rate of the uninsured in this study is considerably smaller than the current proportion of patients who are uninsured in the United States. The study is also limited by the lack of verification of patient perceptions of medical errors by medical record review. Patient recall of medical errors may be affected by physician nonverbal involvement during error disclosures, through which a healing mechanism for physician/patient relationship may occur.^35^ This may have reduced the number of reported errors. Whether the results of this U.S. study apply to other countries requires further analysis. The reliance on pre-enrolled panelists is not a significant limitation given the Internet penetration in the United States and the wide demographic representativeness.

In summary, this large adult U.S. ambulatory care study demonstrates that patient-perceived medical errors are common. The experience of ambulatory care medical errors is more likely perceived for women, those who report poor health, those with limitation of activities, and/or have >2 comorbidities. Higher rates of patient-perceived medical error are more likely among patients who receive care by multiple providers, and among those who utilize more health care resources. These patients require particular monitoring and care.

## Abbreviations Used

AOR: adjusted odds ratio
CI: confidence interval
OR: odds ratio
